# Endoscopic features of the duodenal pyloric gland adenoma: A case series of 14 patients

**DOI:** 10.1002/deo2.70038

**Published:** 2024-11-19

**Authors:** Takeshi Uozumi, Satoru Nonaka, Yasuhiko Mizuguchi, Haruhisa Suzuki, Seiichiro Abe, Shigetaka Yoshinaga, Shigeki Sekine, Yutaka Saito

**Affiliations:** ^1^ Endoscopy Division National Cancer Center Hospital Tokyo Japan; ^2^ Department of Internal Medicine Division of Gastroenterology and Hepatology Nihon University School of Medicine Tokyo Japan; ^3^ Department of Gastroenterology Tokyo Metropolitan Cancer Detection Center Tokyo Japan; ^4^ Division of Diagnostic Pathology National Cancer Center Hospital Tokyo Japan; ^5^ Department of Pathology Keio University School of Medicine Tokyo Japan

**Keywords:** Brunner's gland hyperplasia, duodenum, gastric epithelial metaplasia, pyloric gland adenoma, white opaque substance

## Abstract

**Background:**

Pyloric gland adenoma (PGA) is a distinct subtype of duodenal adenoma. PGA has been increasingly recognized as a histologically and molecularly distinct entity; however, its endoscopic features have not been precisely described. This study aims to investigate the endoscopic characteristics of duodenal PGA, including the association of their putative precursors, Brunner's gland hyperplasia (BGH), and gastric epithelial heterotopia/metaplasia (GEM/H).

**Methods:**

This study was a single‐center, retrospective case series. Fourteen consecutive patients with duodenal PGA were retrieved from the pathological database. PGA was diagnosed according to the World Health Organization classification.

**Results:**

The median tumor size was 22.5 mm (range: 12–40 mm), and 79% of cases were located in the first part of the duodenum. Six PGAs demonstrated high‐grade dysplasia. PGA could be classified into two subtypes based on their appearance: villous lobulated type and smoothly protruding type. BGH and GEM/H were identified in the background mucosa in 28% and 7% of the cases, respectively. BGH was more abundant in the background mucosa of the PGA group than in the control group *(p* < 0.05). Six PGAs (43%) exhibited high‐grade dysplasia, and no significant difference was observed in the endoscopic findings between low‐ and high‐grade dysplasia.

**Conclusions:**

The 14 patients with PGA demonstrated characteristic endoscopic findings. BGH and GEM/H might be precursors of PGA.

## INTRODUCTION

A duodenal adenoma is a rare lesion with a prevalence of less than 0.1%–0.3% in patients undergoing upper gastrointestinal endoscopy.^1^ The World Health Organization (WHO) classification recognizes two distinct subtypes of duodenal adenomas: intestinal‐type adenoma and pyloric gland adenoma (PGA).^2^ Intestinal‐type adenoma is the more common subtype comprising more than 80% of duodenal adenomas.^3^, ^4^ It presents endoscopically as a whitish sessile lesion and is frequently located in the second or third part of the duodenum. With magnifying narrow band imaging (M‐NBI), it demonstrates light blue crest (LBC) and white opaque substance (WOS). ^4^ Histologically, intestinal‐type adenoma exhibits intestinal‐type epithelial differentiation and is comprised of absorptive, goblet, and Paneth cells.^2^, ^3^ Mutation of *APC* is a characteristic genetic feature of intestinal‐type adenoma.^5^

PGA exhibits histological features consistent with pyloric gland differentiation.^6^ Like intestinal‐type adenomas, PGAs frequently harbor *APC* mutations and additionally have activating *GNAS* and *KRAS* mutations.^5^, ^7^ These findings demonstrate that PGA is not only a phenotypically but also a genetically distinct type of duodenal adenoma. PGA often exhibits high‐grade dysplasia and is thought to have a high risk of malignant transformation.^6^, ^8^

Brunner's gland hyperplasia (BGH) and gastric epithelial metaplasia/heterotopia (GEM/H) are non‐neoplastic lesions characterized by a gastric epithelial phenotype and are predominately located in the proximal duodenum.^1^ Notably, GEM/H shares some genetic features with PGAs, including activating *GNAS* and/or *KRAS* mutations.^9^ Based on their common phenotypic features, location, and genetic alterations, previous studies implied that GEM/H might be precursors of PGA. However, the association between these lesions and PGA has not been investigated.^6^

Although PGA has been increasingly recognized as a histologically and molecularly distinct entity, no reports have precisely described the endoscopic features of PGA. This study aims to investigate the endoscopic characteristics of duodenal PGA, including the association of their putative precursors, BGH and GEM/H.

## MATERIALS AND METHODS

### Patients

A consecutive series of patients with duodenal PGA, treated either endoscopically or surgically at the National Cancer Center Hospital between January 2015 and December 2022, were retrieved from the pathological database. We excluded cases with polyposis syndromes such as familial adenomatous polyposis. Finally, we identified 14 duodenal PGAs from 14 patients. The medical records of the patients were reviewed to collect their clinical characteristics, including age, gender, history of *Helicobacter pylori* infection, and endoscopic findings. This study was approved by the Ethics Committee of the National Cancer Center, Tokyo, Japan, and was conducted in accordance with the Declaration of Helsinki. General consents were obtained from all the patients.

### Histological diagnosis

**FIGURE 1:**
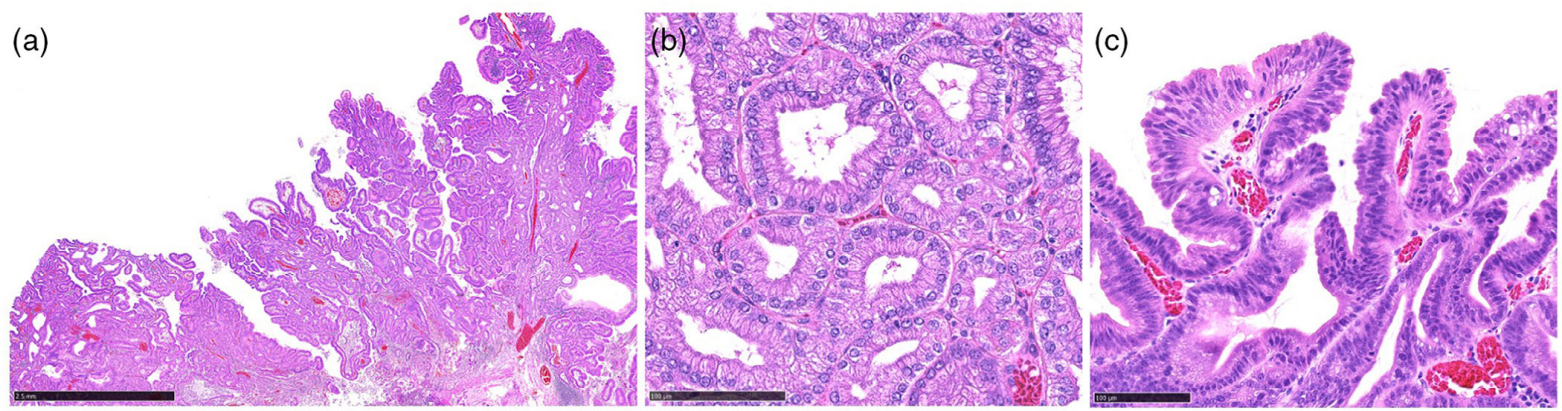
**Histological findings of pyloric gland adenoma**. (a) Villous configuration and some dilated glands were observed with low‐power magnification (Black scale bar is 2.5 mm). (b) High‐power magnification reveals the characteristic ground‐glass cytoplasm and basal round nuclei (Black scale bar is 100µm). (c) Covered with foveolar differentiated epithelium with mild atypia (Black scale bar is 100µm).

PGA was diagnosed according to the WHO classification.^2^ PGA consists of closely packed pyloric‐type glands lined by cuboidal to low columnar cells. The tumor cells have round and basally located nuclei with inconspicuous nucleoli (Figure 1). High‐grade dysplasia is characterized by increased architectural complexity and/or marked cytological atypia. The adenomas displaying more than 10% high‐grade dysplasia were diagnosed as high‐grade lesions.^5^ Histopathological diagnoses were confirmed by a pathologist (Shigeki Sekine) specializing in gastrointestinal pathology.

### Endoscopic procedures and image analysis

Preoperative diagnostic endoscopy was performed with high‐resolution endoscopy (GIF‐H290, GIF‐H290ZI, or GIF‐XZ1200; Olympus) and endoscopy system (EVIS LUCERA SPECTRUM or EVIS X1; Olympus). The video processor settings were as follows: the structure enhancement was set at the B6 level for white light imaging (WLI) and B8 level for narrow band imaging (NBI), and the color enhancement was set at the Cm1 level. Chromoendoscopy with indigo carmine was routinely performed for diagnostic endoscopy. The endoscopic findings, including size, color, macroscopic type, location, and magnifying endoscopic findings, were immediately reported after examination and stored in the endoscopic image server database (Nexsus; Fujifilm). Two expert endoscopists (Takeshi Uozumi and Satoru Nonaka), board‐certified fellows of the Japanese Gastroenterological Endoscopy Society, reviewed the stored endoscopic images and reports. In case of discordance in the endoscopic findings among the endoscopists, consensus was reached through discussion.

### Definition of the endoscopic findings

The macroscopic type and surface structure were evaluated with indigo carmine sprayed images. The macroscopic type was assessed according to the Paris endoscopic classification^10^ and divided into two groups: protruding (type 0‐Ip or 0‐Is) and non‐protruding (type 0‐IIa or 0‐IIc). The tumor coloration was assessed based on the predominant color tone. The presence of distinctly white mucosa, characterized by well‐defined surface structures with a white substance, was also evaluated.^11^

The presence of LBC^12^ and oval‐shaped marginal epithelium (OME)^4^ were evaluated with M‐NBI. The definitions of the LBC and OME were: LBC is a bright, blue‐white line on the crests of the epithelial surface/gyri^12^ (Figure S1a); OME is a curved or oval‐shaped white rim surrounding the glandular or villous structures, resembling the white zone^13^ in the gastric epithelium (Figure S1b).

### Background mucosa

The presence of BGH and GEM/H was evaluated endoscopically. BGH was defined as a gently rising submucosal tumor‐like lesion, occasionally accompanied by orifices^1^, ^14^ (Figure S1c). GEM/H was defined as a single or multiple, sessile lesion(s) in the duodenal bulb mimicking the gastric epithelium, displaying a distinct demarcation from the surrounding duodenal villi (Figure S1d)^1^. To compare the prevalence of those non‐neoplastic lesions between the PGA and control groups, we reviewed the screening or surveillance endoscopies performed in November 2023 as a control group. The following cases that could not be assessed for the duodenum were excluded from the control group: the cases with a history of surgery, radiation, or chemotherapy for the upper gastrointestinal tract, familial adenomatous polyposis, or lack of images of the duodenal bulb on endoscopy. Among the 883 consecutive cases that underwent screening or surveillance upper gastrointestinal endoscopy in November 2023, a total of 517 cases were manually reviewed as the control group.

### Immunohistochemical staining

Immunohistochemical staining was performed by an automated stainer (Dako; Glostrup) in accordance with the manufacturer's protocol. To evaluate the phenotypic expression, the following antibodies were used: MUC5AC (CLH2; 1:200 Norvocastra Laboratories), MUC6 (CLH5; 1:200, Santa Cruz Biotechnology), MUC2 (Ccp58; 1:200, Norvocastra Laboratories), and CD10 (56C6, 1:100, Norvocastra). Lesions with more than 20% positive cells were regarded as positive.^5^ We also used an anti‐adipophilin antibody (AP125; 1:100; Acris Antibodies, Inc.) to evaluate the association between the distinctly white mucosa and lipid droplets. The degree of adipophilin staining was evaluated as <1%, 1%–10%, or >10% of superficial epithelial cells stained with anti‐adipophilin antibody.

### Statistical analysis

Fisher's exact test and Wilcoxon's rank‐sum test were used for categorical and continuous variables as appropriate. *p* < 0.05 was considered statistically significant. Statistical analysis was performed using the EZR software program.^15^

## RESULTS

### Clinicopathological features of patients with duodenal PGA

The median age was 67 years (range, 60–84 years), and the majority of the patients were male (79％ [11/14]). Although half of the patients had a history of *H. pylori* infection, open‐type atrophic gastritis was rare (14% [2/14]). PGA was positive for the gastric epithelial markers; MUC6 (100% [14/14]) and MUC5AC (93% [13/14]) and negative for intestinal epithelial markers; MUC2 and CD10. Six PGAs exhibited high‐grade dysplasia (43% [6/14]; Table 1).

**TABLE 1:** Clinicopathological features of the 14 pyloric gland adenomas.

	All (*n* = 14)	Villous lobulated type (*n* = 10)	Smoothly protruding type (*n* = 4)
**Patient characteristics**			
Age, median [range]	67 [60–84]	66 [60–84]	71 [62–75]
Sex, male/female	11/3	7/3	4/0
**Past History of** *H. pylori* **infection** **Positive/negative/not available**	7/1/6	4/1/5	3/0/1
**Gastric atrophy** ^a^ **Closed type/open type/not available**	11/2/1	8/1/1	3/1/0
**Endoscopic findings**			
Size, median [range]	22.5 [12–40]	25 [12–40]	12 [12–25]
Location, first/second part	11/3	10/0	1/3
Macroscopic appearance, protruding/non‐protruding	11/3	7/3	4/0
Color, reddish/pale	4/10	2/8	2/2
Distinctly white mucosa, present/absent	4/10	4/6	0/4
BGH, present/absent	4/10	2/8	2/2
GEM/H, present/absent	1/13	1/9	0/4
**M‐NBI**			
OME, present/absent	8/1	5/1	3/0
LBC, present/absent	0/9	0/6	0/3
**Histopathology**			
Low‐grade dysplasia/high‐grade dysplasia	8/6	6/4	2/2
**Immunohistochemical staining**			
MUC5AC, positive/negative	13/1	9/1	4/0
MUC6, positive/negative	14/0	10/0	4/0
MUC2, positive/negative	0/14	0/10	0/4
CD10, positive/negative	0/14	0/10	0/4
Adipophilin, <1%/1%–10%/>10%	5/4/5	3/2/5	2/2/0

^a^ Kimura Takemoto classification.

Abbreviations: BGH, Brunner's gland hyperplasia; GEM/H, gastrid epithelial hyperplasia; *H. pylori*, *Helicobacter pylori*; LBC, light blue crest; M‐NBI, magnifying narrow band imaging; OME, oval‐shaped marginal epithelium.

### Endoscopic characteristics of duodenal PGA

The median tumor size was 22.5 mm (range: 12–40 mm), and 11 cases (79%) were located in the first part of the duodenum. The macroscopic type was protruding in 11 cases (79%). The coloration was pale in 10 cases (71%), and distinctly white mucosa was observed in four cases (28%). In the background mucosa, BGH and GEM/H were identified in four (28%) and one (7%) cases. M‐NBI was performed for nine cases. OME was observed in 8 cases (89%), although LBC was not detected in any of the cases (Table 1).

PGA could be classified into two subtypes based on their appearance: villous lobulated type and smoothly protruding type.

### Villous lobulated type

**FIGURE 2:**
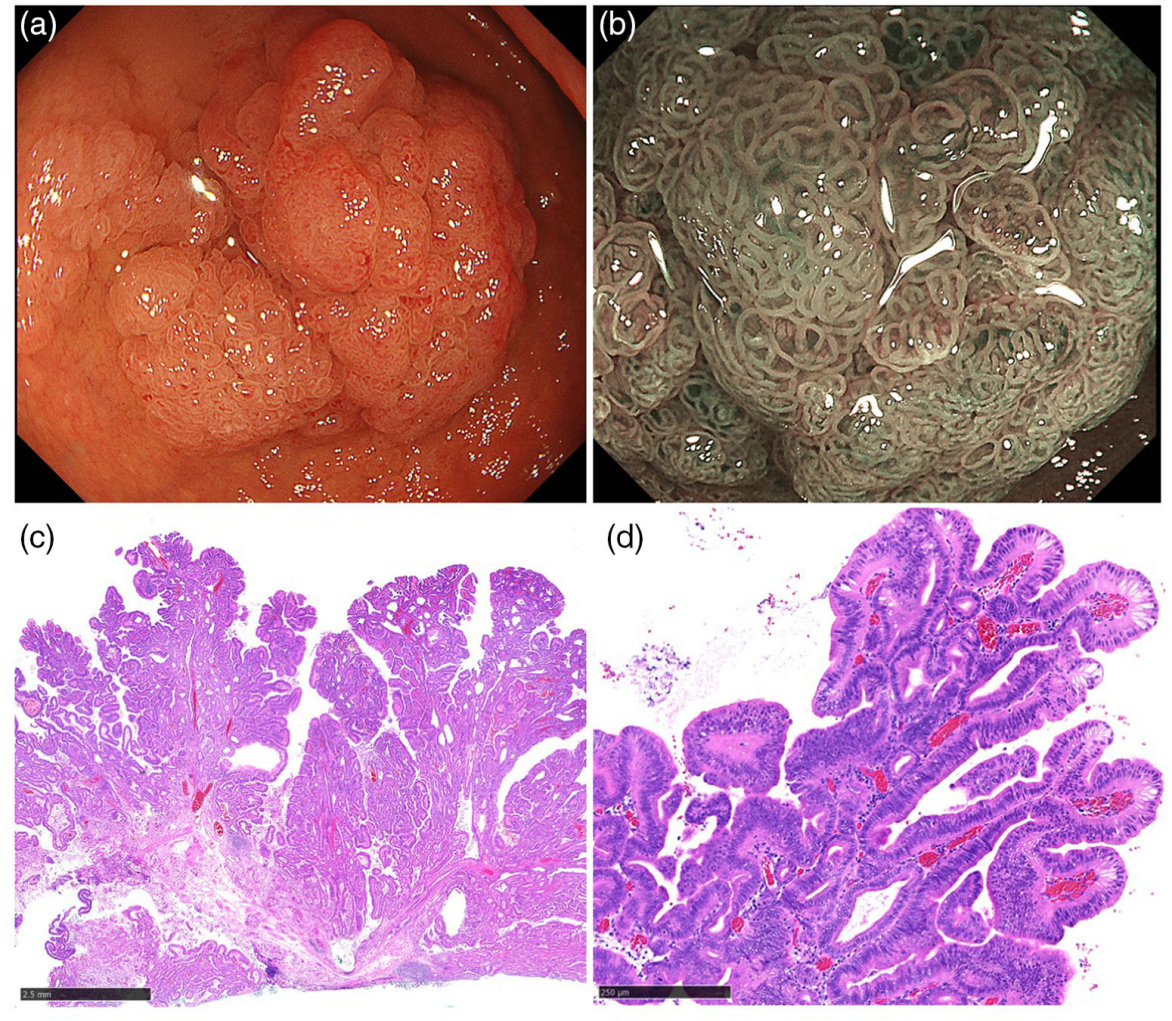
**Villous lobulated pyloric gland adenoma**. (a) A pale color, protruding lesion with villous or lobulated surface structure. (b) Magnifying narrow‐band imaging reveals oval‐shaped marginal epithelium on the surface mucosa. (c) Villous and lobulated configurations were observed with low power magnification (Black bar is 2.5 mm). (d) Foveolar differentiated epithelium with a finger‐like tall structure (Black scale bar is 250µm).

Villous lobulated type was the most common appearance (71% [10/14]). This type was characterized by a villous or lobulated surface structure. The macroscopic type was protruding in seven cases, and non‐protruding in three cases. The median size was 25 mm (range: 12–40 mm), and all were in the first part of the duodenum. The coloration was pale in eight cases (80%), and distinctly white mucosa was observed in four cases (40%). M‐NBI was performed for six cases, and five cases demonstrated OME. Histologically, a villous or lobulated configuration with some dilated glands was observed regardless of their macroscopic type. Foveolar differentiated epithelium with a finger‐like tall structure was observed on the surface. High‐grade dysplasia was detected in four cases (40%). Representative endoscopic and histological images of the villous lobulated type are demonstrated in Figure 2.

### Smoothly protruding type

**FIGURE 3:**
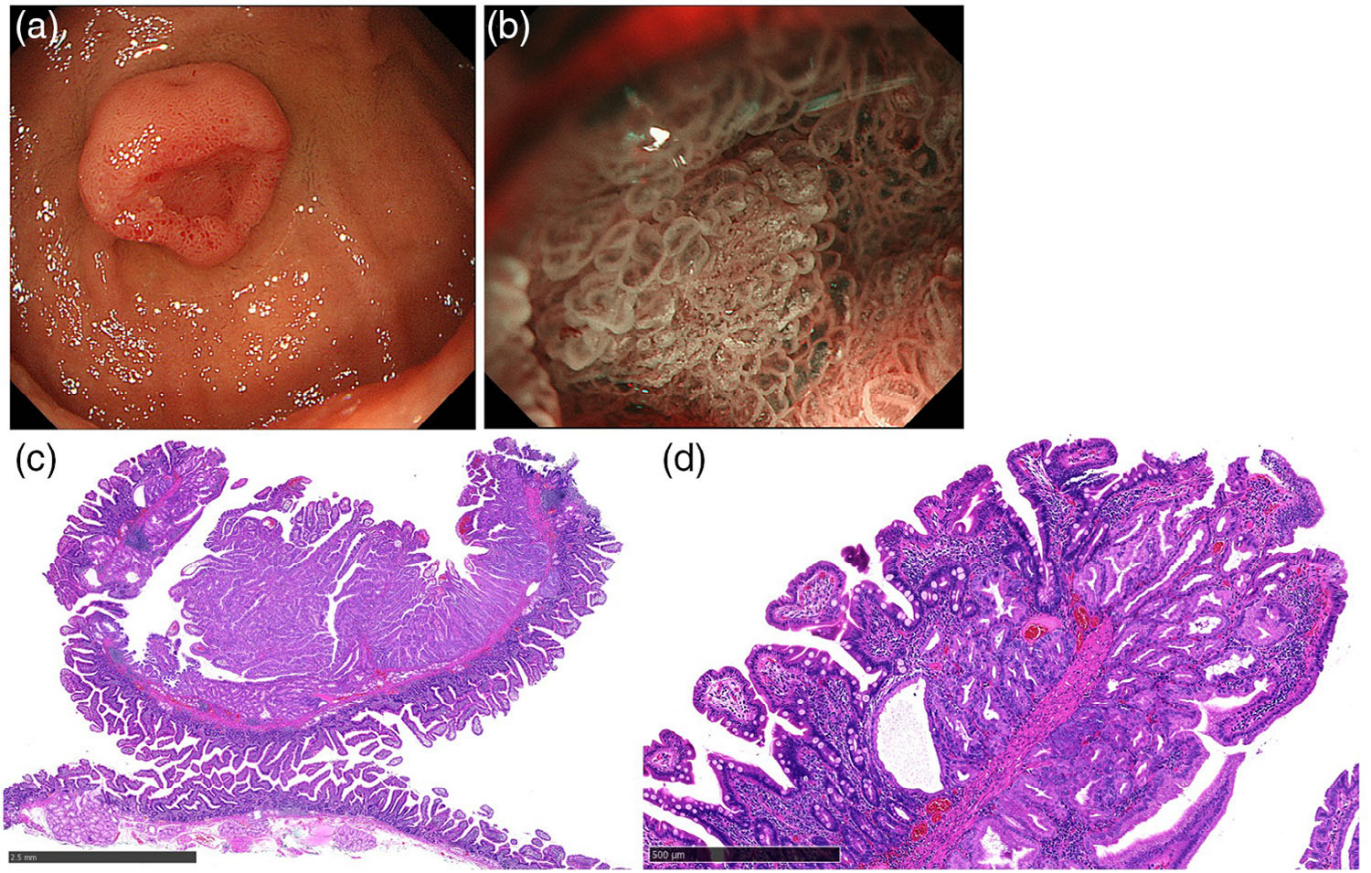
**Smoothly protruding pyloric gland adenoma**. (a) A submucosal tumor‐like protruding lesion with central depression. (b) Magnifying narrow‐band imaging shows oval‐shaped marginal epithelium at the central depression. (c) Low‐power magnification reveals finger‐like tall structures at the depressed area (Black bar is 2.5 mm). (d) The smooth protrusion was covered with duodenal epithelium and foveolar differentiated epithelium was observed inside the depressed area (Black bar is 500 µm).

Four cases demonstrated this appearance (29% [4/14]). The smoothly protruding type was characterized by a submucosal tumor‐like protrusion with a central depression. The median size was 12 mm (range: 12–25 mm), and three were located in the second part of the duodenum (75%). In three cases where M‐NBI was performed, all demonstrated OME at the central depression. Histologically, the smooth protrusion was covered with duodenal epithelium. Foveolar differentiated epithelium with a finger‐like tall structure was observed in the depressed area. High‐grade dysplasia was detected in two cases (50%). Representative endoscopic and histological images of the smoothly protruding type are demonstrated in Figure 3.

Detailed clinicopathological features and endoscopic findings of the 14 PGAs are presented in Table S1.

### Relationships between OME and histological findings

M‐NBI was performed in nine cases, meanwhile, OME was observed in eight cases. Histologically, all cases had foveolar differentiated epithelium with a finger‐like tall structure on the surface. However, this surface structure was less common in one case which was negative for OME. For further evaluation, we compared the endoscopic and histological findings in one case that was suitable for one‐to‐one matching evaluation. With a maximum magnification using GIF‐H290Z, OMEs measure 200–400 µm in diameter. Histologically, the foveolar differentiated finger‐like tall structures were about 150–300 µm, consistent with OME in size (Figure S2).

### Background mucosa

The median age was 69 years (range 24–93 years), and 331 cases were male (64%) in the control group. BGH and GEM/H were observed in four (0.77%) and 12 (2.3%) cases in the control group, respectively. All BGHs were located in the second part of the duodenum, on the oral side of the papilla. All cases of GEM/H were observed in the first part of the duodenum. The indications for endoscopy in the control group are presented in Figure S3. The prevalence of BGH was significantly higher in the PGA group than in the control group (0.77% vs. 28%, *p* < 0.05). However, no statistically significant difference was observed in the prevalence of GEM/H between the two groups (2.3% vs. 7%, *p =* 0.296).

### Relationships between the distinctly white mucosa and adipophilin staining

**FIGURE 4:**
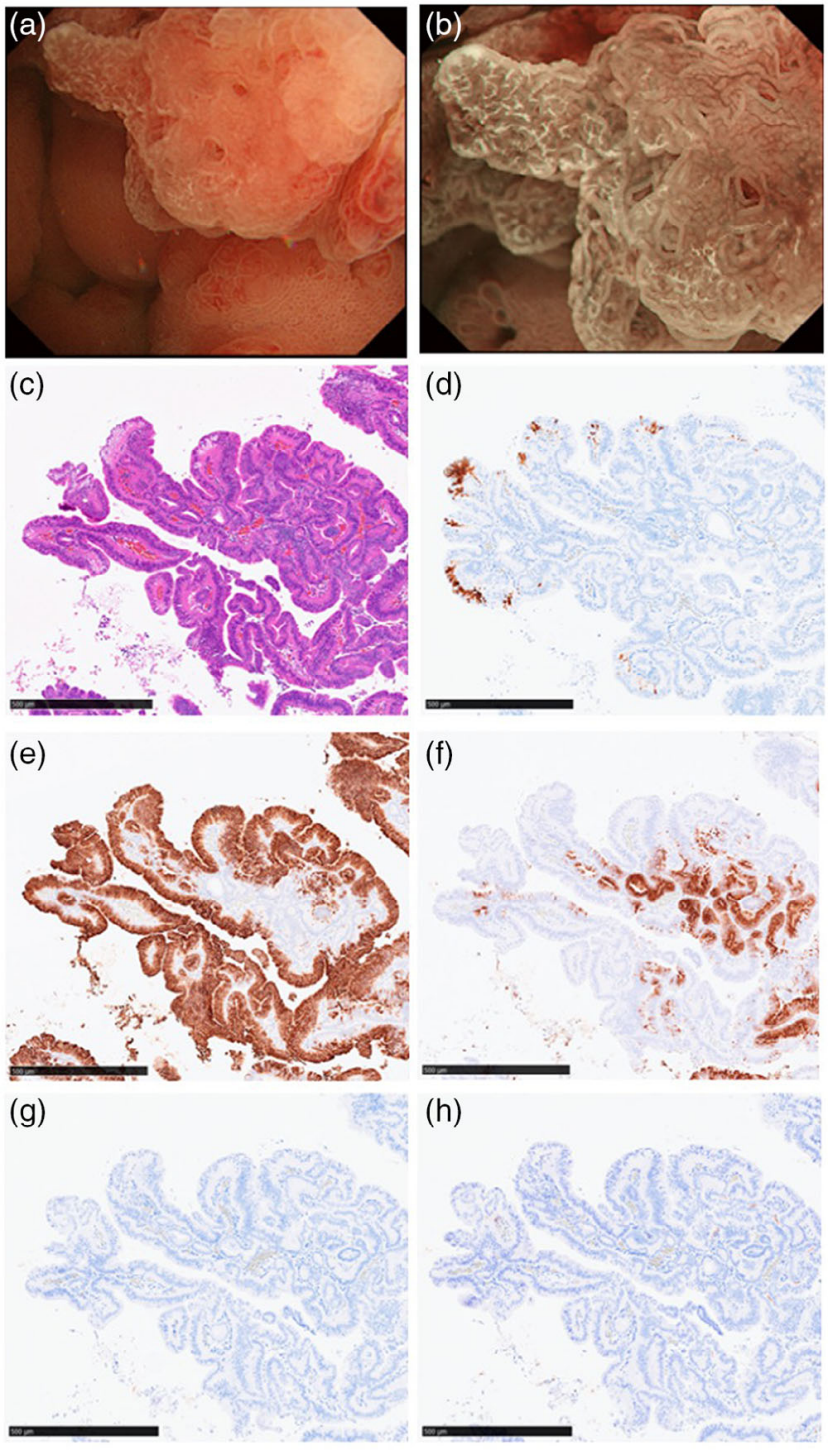
**Endoscopic and histopathological characteristics of distinctly white mucosa**. (a) The distinctly white mucosa was characterized by well‐defined surface structures with a white substance. (b) The white substance became clearer with magnifying narrow‐band imaging. (c) Hematoxylin and eosin staining. (d) Adipophilin staining. (e) MUC5AC. (f) MUC6. (g) MUC2. (h) CD10.

Immunohistochemical staining revealed nine cases of PGA (64.3%) were positive for adipophilin staining in at least 1% of the superficial epithelium. With M‐NBI, distinctly white mucosa was noted in four cases (28%), all of which were adipophilin positive, exhibiting more than 1% staining in the superficial epithelium. (Table 2). The adipophilin‐positive epithelium was positive for the MUC5AC and negative for the MUC6, MUC2, and CD10 (Figure 4).

**TABLE 2:** The relationships between distinctly white mucosa and adipophilin staining.

	Distinctly white mucosa		
	Positive (*n* = 4)	Negative (*n* = 10)	
**Adipophilin**			*p* = 0.131
<1%	0	5	
1%–10%	1	3	
10%<	3	2	

### Comparison between high‐grade dysplasia and low‐grade dysplasia

There were six cases (43%) of high‐grade dysplasia. Endoscopic and clinicopathological findings were compared between low‐ and high‐grade dysplasia, and there were no significant differences (Table 3).

**TABLE 3:** Comparison of low‐ and high‐grade dysplasia.

	Low grade (*n* = 8)	High grade (*n* = 6)	*p‐*value
**Patient's characteristics**			
Age, median [range]	67 [60–75]	68 [62–84]	0.7
Sex, male/female	7/1	4/2	0.54
**Endoscopic findings**			
Size, median [range]	20 [12–40]	27 [12–40]	0.47
Location, first/second part	6/2	5/1	1
Macroscopic appearance, protruding/non‐protruding	7/1	4/2	0.54
Color, red/pale	1/7	3/3	0.25
White mucosa, present/absent	3/5	1/5	0.58
BGH, present/absent	3/5	1/5	0.58
GEM/H, present/absent	0/7	1/6	1
**M‐NBI**			
OME, present/absent	5/1	3/0	1
LBC, present/absent	0/6	0/3	1
**Immunohistochemical staining**			
Adipophilin, <1%/1%–10%/>10%	3/1/4	2/3/1	0.33

Abbreviations: BGH, Brunner's gland hyperplasia; GEM/H, gastrid epithelial hyperplasia; LBC, light blue crest; M‐NBI, magnifying narrow band imaging; OME, oval‐shaped marginal epithelium.

## DISCUSSION

PGA is thought to be associated with a high risk of malignant transformation, and endoscopic resection is recommended.^6^, ^8^ Although biopsy is the gold standard for definitive diagnosis, it can cause submucosal fibrosis and make subsequent endoscopic resection challenging.^16^ Therefore, diagnosing PGA without biopsy is desirable. To achieve a biopsy‐independent diagnosis, knowledge of both endoscopic and histological characteristics is crucial. We described the endoscopic and histological findings of PGA precisely; thus, our study will be helpful in diagnosing PGA visually. In this case series, most PGAs were pale in color, with protruding lesions having villous or lobulated surface structures. However, the four PGAs demonstrated a submucosal tumor‐like appearance and were obviously different from the others. This type should be distinguished from the other PGAs as it can be misdiagnosed as a submucosal tumor or non‐neoplastic lesion, such as BGH. Therefore, we classified PGA into two subtypes based on their appearance: villous lobulated type and smoothly protruding type.

OME was a characteristic finding of PGA. Histologically, OME was consistent with foveolar differentiated epithelium with a finger‐like tall structure. Recently, Nakayama et al. proposed a new diagnostic algorithm using M‐NBI for differentiating gastric‐type duodenal neoplasms from non‐neoplastic lesions.^17^ This algorithm uses a closed‐loop structure (consistent with OME) with enlarged marginal epithelium and a demarcation line.^17^ By applying this algorithm to our cases, we were able to accurately diagnose even the smoothly protruding type as neoplastic lesions. M‐NBI might be useful for detailed endoscopic diagnosis.

This study revealed that BGH existed more frequently in the background mucosa of the PGA group than in that of the control group. Previous genetic analyses have demonstrated that GEM/H might be a precursor of the PGA^9^; however, GEM/H was not as common as BGH in this study. BGH occurs in the lamina propria or the submucosal layer. Thus, an accurate diagnosis of BGH can be challenging solely based on biopsy specimens.^1^ Moreover, BGH is sometimes covered with GEM/H; therefore, BGH could be misdiagnosed as GEM/H if the biopsy specimen is insufficient. In the previous study,^9^ biopsy specimens used for genetic analysis may have contained BGH to some extent. Considering the results of this study and previous genetic analyses, not only GEM/H but also BGH covered with GEM/H may be precursors of PGA.

In this study, the distinctly white mucosa was positive for adipophilin staining, a histological biomarker for lipid droplets.^18^ This observation implies that distinctly white mucosa is consistent with WOS both endoscopically and histologically. There are two possible mechanisms of lipid droplet accumulation: the absorption hypothesis and the production hypothesis.^19^ The absorption hypothesis is supported by the fact that the fat loading test increases lipid droplets in the epithelium.^20^ Ingested lipids are absorbed into the epithelium via absorptive cells^20^; therefore, WOS has been believed to be a specific finding of intestinal‐type mucosa.^19^, ^21^ However, our study showed that lipid droplets can accumulate in the gastric‐type epithelium as well. The production hypothesis, suggests that lipid droplets are synthesized from glucose and fatty acids supplied by capillaries, which may explain lipid accumulation in the gastric‐type epithelium. The detailed mechanism of lipid droplet accumulation has not yet been elucidated, and further studies are necessary.

PGAs with high‐grade dysplasia were identified in 42% of the PGA in this study, which is consistent with the findings of previous reports.^6^ High‐grade dysplasia is considered to be a risk factor for malignant transformation; thus, it is important to differentiate low‐ and high‐grade dysplasia. This study revealed no significant differences in clinicopathological features between the two groups.

This study had certain limitations. First, this study did not include PGAs diagnosed by biopsy specimens. Second, most PGAs were resected with piecemeal endoscopic mucosal resection, resulting in inappropriate one‐to‐one matching of the endoscopic images and histological findings. Third, the control group for measuring the prevalence of BGH and GEM/H was not a healthy population. It should be noted that the control group was mainly composed of surveillance endoscopy after endoscopic resection for esophageal or gastric cancer. Fourth, we could not investigate the relationships between *H. pylori* infection and duodenal PGAs. *H. pylori* infection can cause inflammation and peptic ulcers in the duodenal bulb, leading to gastric epithelial metaplasia. Considering GEM/H might be a precursor of duodenal PGA, *H. pylori* infection can affect the incidence of duodenal PGA. However, our data was insufficient to discuss it due to a lack of information about the history of *H. pylori* infection status. Lastly, M‐NBI was not performed in five cases. Even with these limitations, this study has value because no prior reports have investigated the endoscopic findings of duodenal PGA.

In conclusion, the 14 patients with PGA demonstrated characteristic endoscopic findings. BGH and GEM/H might be precursors of PGA.

## CONFLICT OF INTEREST STATEMENT

Seiichiro Abe is an Associate Editor in DEN Open.

## ETHICS STATEMENT

This retrospective study was approved by the Institutional Reviewer Board (IRB number: 2016–447).

## PATIENT CONSENT STATEMENT

Comprehensive consents for the retrospective study were obtained from all the patients.

## Supporting information



Table S1 Detailed clinicopathological features of the 14 PGAs.

Figure S1 Representative endoscopic images of LBC, OME, BGH, and GEM/H. (a) Yellow arrows show LBC. (b) Yellow arrows show OME. (c) BGH. (d) GEM/H.

Figure S2 One‐to‐one matching between OME and foveolar differentiated finger‐like tall structure. (a) Maximum magnification using GIF‐H290ZI. The object appearing on the entire screen is about 4.75 mm (one square is 1 × 1 mm). (b) Maximum magnification focusing on the central depression. This endoscopic image was divided into 15 × 15 squares by a grid line, so one square is about 300 µm. The size of each OME was around one square. (c) The Black scale bar is 250 µm. The diameter of the finger‐like tall structure is 150–300 µm.

Figure S3 Flow chart of the control group.
