# Supplementary material for: Endoscopic features of the duodenal pyloric gland adenoma: A case series of 14 patients
Source: DEN Open. 2024 Nov 19;5(1):e70038. doi: 10.1002/deo2.70038 (PMC11579376; doi:10.1002/deo2.70038)
Supplement: Supplementary file 1 — Table S1 Detailed clinicopathological features of the 14 PGAs. [file DEO2-5-e70038-s001.docx]

**Supplementary table1. Detailed clinicopathological features of the 14 PGAs**

| Case | Age | Sex | Historyof H.pylori infection | Gastric atrophy | Location | Size | Pari's classification | Macroscopic type | Color | Macroscopic appearance | Background | Distinctly white mucosa | LBC | OME | Grade | Adipophilin |
| --- | --- | --- | --- | --- | --- | --- | --- | --- | --- | --- | --- | --- | --- | --- | --- | --- |
| DPGA01 | 84 | F | N/A | C0 | first part | 25 | IIa | non-protruding | pale | Villous lobulated | | None |  |  | High | 1-10% |
| DPGA02 | 66 | M | N/A | C0 | first part | 28 | Is | protruding | pale | Villous lobulated | | None |  |  | High | <1% |
| DPGA03 | 75 | M | N/A | C0 | second part | 25 | Is+IIc | protruding | pale | Smoothly protruding | BGH | None | - | + | Low | <1% |
| DPGA04 | 62 | M | Positive | C2 | second part | 12 | Is+IIc | protruding | reddish | Smoothly protruding | BGH | None | - | + | High | 1-10% |
| DPGA05 | 65 | M | N/A | C0 | first part | 40 | IIa | non-protruding | reddish | Villous lobulated | GHM/H | None | - | + | High | <1% |
| DPGA06 | 60 | F | Positive | C3 | first part | 20 | Is | protruding | pale | Villous lobulated | | Exist |  |  | Low | 10%< |
| DPGA07 | 62 | M | Positive | C2 | first part | 20 | Ip | protruding (pedunculated) | pale | Villous lobulated | | Exist | - | - | Low | 10%< |
| DPGA08 | 69 | F | N/A | O1 | first part | 35 | Is | protruding | pale | Villous lobulated | | Exist |  |  | High | 10%< |
| DPGA09 | 69 | M | Positive | O3 | first part | 12 | Is+IIc | protruding | reddish | Smoothly protruding | | None | - | + | High | 1-10% |
| DPGA10 | 61 | M | N/A | C0 | first part | 16 | IIa | non-protruding | pale | Villous lobulated | BGH | None | - | + | Low | <1% |
| DPGA11 | 73 | M | Positive | C3 | second part | 12 | Is+IIc | protruding | pale | Smoothly protruding | | None |  |  | Low | <1% |
| DPGA12 | 66 | M | Positive | C2 | first part | 25 | Is | protruding | pale | Villous lobulated | BGH | Exist | - | + | Low | 1-10% |
| DPGA13 | 70 | M | Negative | C0 | first part | 40 | Is | protruding | reddish | Villous lobulated | | None | - | + | Low | 10%< |
| DPGA14 | 68 | M | Positive | N/A^※^ | first part | 12 | Is | protruding | pale | Villous lobulated | | None | - | + | Low | 10%< |

※Remnant stomach (Post proximal gastrectomy for gastric cancer.)
