# Supplementary figures and images for: Endoscopic features of the duodenal pyloric gland adenoma: A case series of 14 patients
Source: DEN Open. 2024 Nov 19;5(1):e70038. doi: 10.1002/deo2.70038 (PMC11579376; doi:10.1002/deo2.70038)

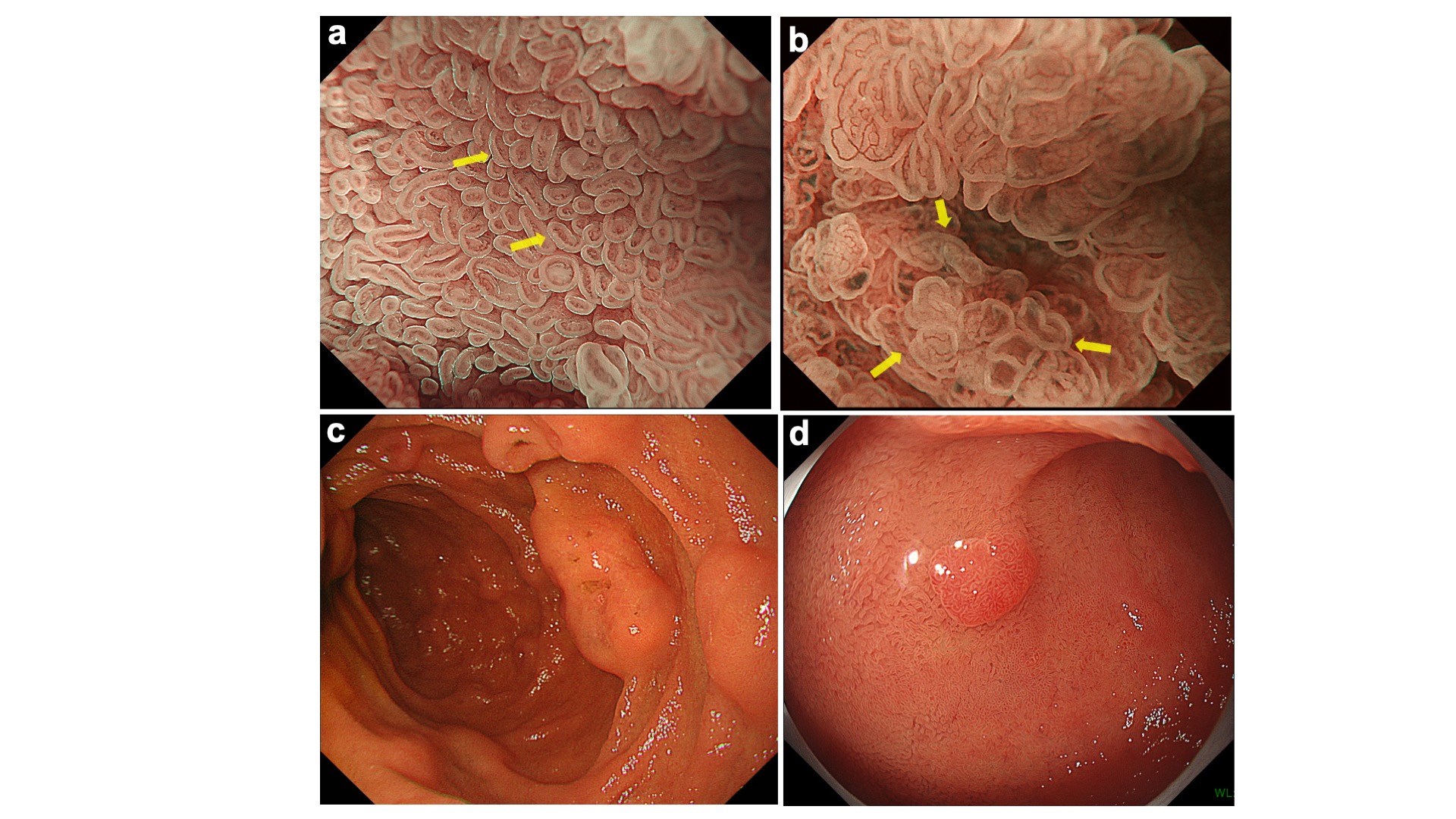

Supplement: Supplementary file 2 — Figure S1 Representative endoscopic images of LBC, OME, BGH, and GEM/H. (a) Yellow arrows show LBC. (b) Yellow arrows show OME. (c) BGH. (d) GEM/H. [file DEO2-5-e70038-s004.jpeg]

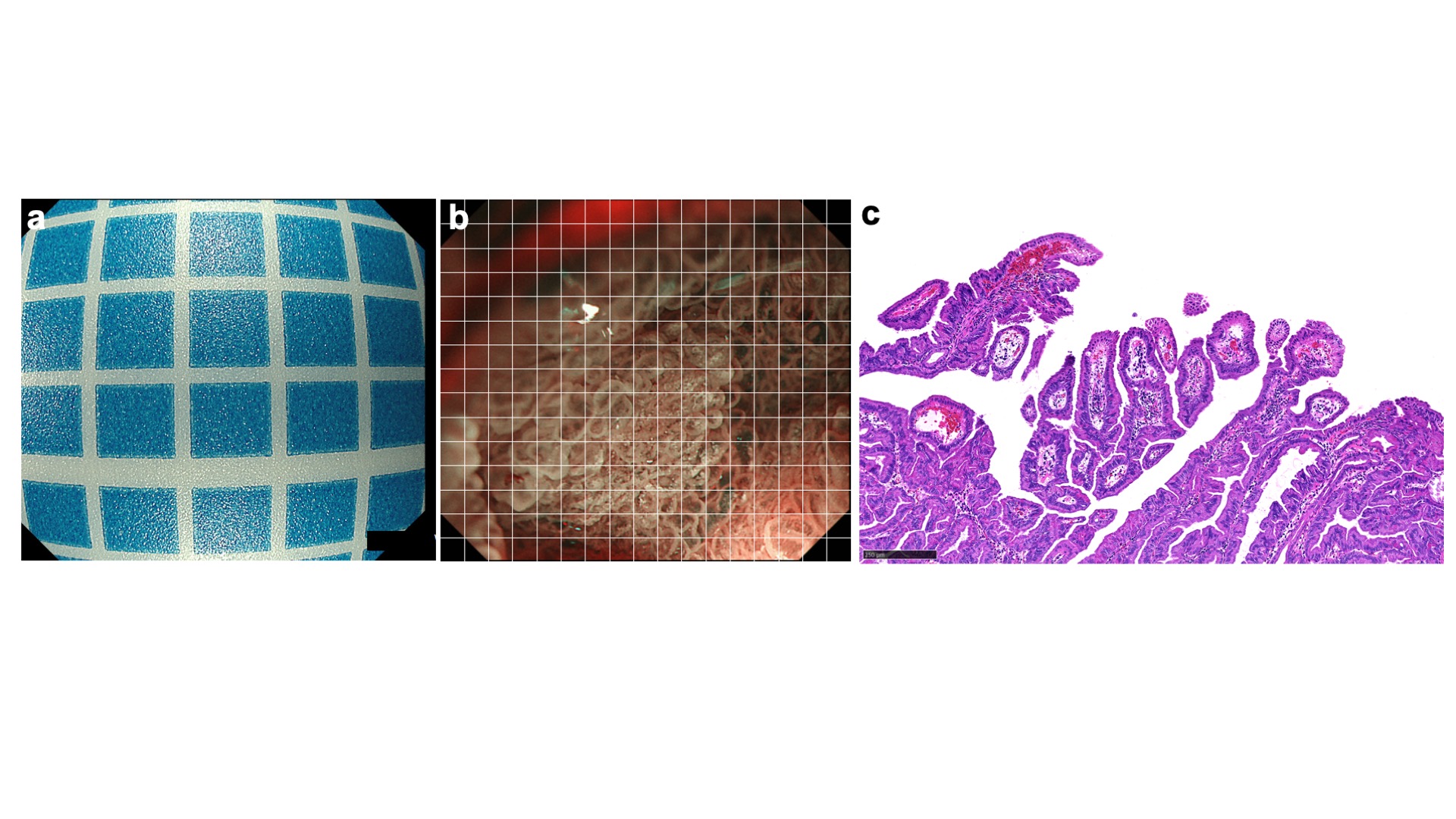

Supplement: Supplementary file 3 — Figure S2 One‐to‐one matching between OME and foveolar differentiated finger‐like tall structure. (a) Maximum magnification using GIF‐H290ZI. The object appearing on the entire screen is about 4.75 mm (one square is 1 × 1 mm). (b) Maximum magnification focusing on the central depression. This endoscopic image was divided into 15 × 15 squares by a grid line, so one square is about 300 µm. The size of each OME was around one square. (c) The Black scale bar is 250 µm. The diameter of the finger‐like tall structure is 150–300 µm. [file DEO2-5-e70038-s002.jpeg]

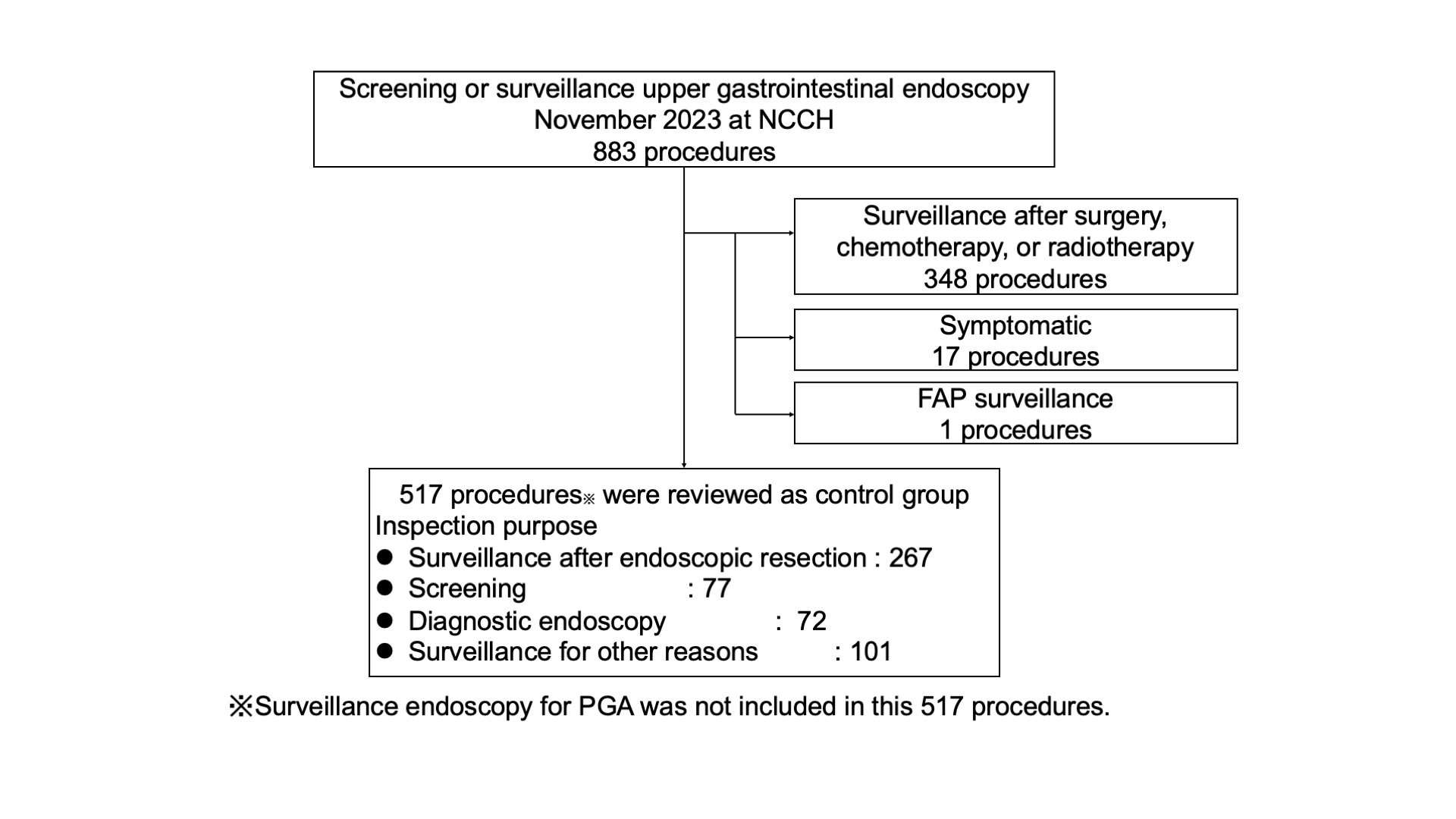

Supplement: Supplementary file 4 — Figure S3 Flow chart of the control group. [file DEO2-5-e70038-s003.jpeg]
